# A thermodynamic investigation into protein–excipient interactions involving different grades of polysorbate 20 and 80

**DOI:** 10.1007/s10973-024-13533-6

**Published:** 2024-08-25

**Authors:** Joseph Whiteley, Laura J. Waters, James Humphrey, Steve Mellor

**Affiliations:** 1 School of Applied Sciences, University of Huddersfield, Queensgate, Huddersfield HD1 3DH UK; 2 Croda Europe Ltd, Cowick Hall, Snaith, Goole DN14 9AA UK

**Keywords:** Biopharmaceutical, Critical micellar concentration, Protein aggregation, Stability, Surfactants

## Abstract

Developing stable biopharmaceutical formulations is of paramount importance and is typically achieved by incorporating surfactants as stabilising agents, such as polysorbate 20 and 80. However, little is known about the effect surfactant grade has on formulation stability. This study evaluates the effect of regular grade and Super-refined™ polysorbates 20 and 80 and their interaction with model proteins, namely β-lactoglobulin (β-Ig), human serum albumin (HSA) and immunoglobulin gamma (IgG), using isothermal titration calorimetry (ITC) and differential scanning calorimetry (DSC). ITC results indicated that all four polysorbates underwent binding interactions with β-Ig and HSA, yet no interaction was observed with IgG this is postulated to be a consequence of differences in secondary structure composition. Surfactant binding to β-Ig occurred at ratios of ~ 3:2 regardless of the surfactant used with dissociation constants ranging from 284 to 388 µM, whereas HSA bound at ratios of ~ 3:1 and dissociation constants ranging from 429 to 653 µM. Changes in enthalpy were larger for the surfactant interactions with HSA compared with β-Ig implying the former produced a greater binding interaction than the latter. DSC facilitated measurement of the temperature of unfolding of each protein with the presence of each polysorbate where results further confirmed interactions had occurred for β-Ig and HSA with an increased unfolding temperature between 4 and 6 K implying improved protein stability, yet again, no interaction was observed with IgG. This study thermodynamically characterised the role of polysorbates in protein stabilisation for biopharmaceutical formulations.

## Introduction

**Fig. 1:**
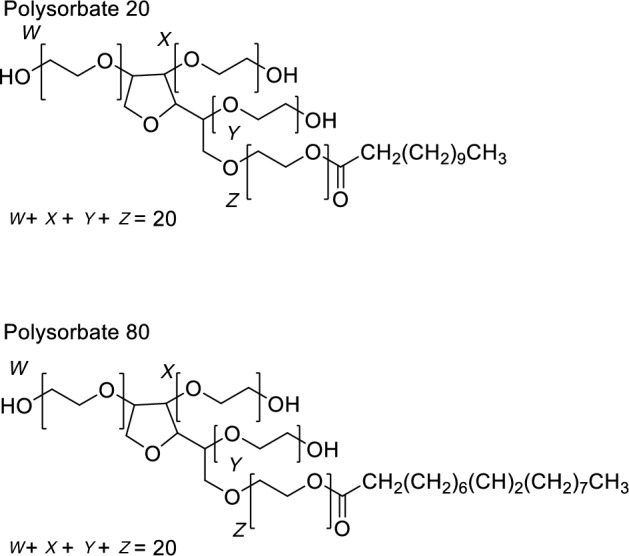
Tween™ 20 (top) and Tween™ 80 (bottom) [11]

Biopharmaceuticals, also known as biologics, are becoming the most effective method for handling conventionally difficult to treat diseases such as cancer and Alzheimer's disease [1]. These drugs are usually delivered to patients using parental routes of administration [2], with monoclonal antibodies (mAbs) being the most frequently used system [3]. However, it has been well documented that biologics are incredibly susceptible to degradation via several routes including chemical changes to constituent amino acids, as well as physical changes to proteins in the form of aggregation and unfolding [4–6]. Physical degradation is a common problem in the formulation stage of biologic development and tends to result in the formation of oligomers [7]. This occurs via several different mechanisms including reversible oligomerisation of native proteins [8], aggregation due to chemical changes [9] and adsorption of proteins on to the surfaces of containers causing changes in protein structure which leads to aggregation [7]. Both reversible oligomerisation and irreversible oligomerisation, in addition to surface-induced aggregation, are problems that can occur regardless of the stability of the protein molecules themselves. Overcoming these susceptibilities to produce a stable formulation can be achieved by adding excipients such as surfactants [10]. The most common surfactants in use are polysorbates 20 and 80 (Fig. 1), available as Tween™ 20 and Tween™ 80 by Croda Ltd. [11]. Polysorbate 20 and polysorbate 80 are used due to their non-ionic nature and low toxicity [12]. Their structure consists of a sorbitan molecule that has undergone esterification with polyoxyethylene (POE) and then further esterification of the POE chains with lauric acid for polysorbate 20 and oleic acid for polysorbate 80 [13, 14].

It has been shown that polysorbate 20 and polysorbate 80 are often included in biologic formulations at concentrations of around 0.11 mM and 0.19 mM, respectively, ten times lower than the critical micellar concentration (CMC) reported in a recent study [15]. Surfactants stabilise biologic formulations against surface-induced aggregation via displacement of proteins at interfaces by preferential competitive adsorption [16, 17]. In order to stabilise against reversible oligomerisation of native proteins, surfactant molecules interrupt bonding interactions of proteins; this type of interaction has been investigated using various different proteins as model biologics, including bovine serum albumin (BSA) and different surfactants, such as dodecyltrimethylammonium bromide (DTAB) [18–20]. Investigations into proteins and surfactant protein interactions are often conducted using differential scanning calorimetry (DSC) [21–26] and isothermal titration calorimetry (ITC) [18, 27]. *Garidel *et al*.* have investigated the interactions of human serum albumin and immunoglobulins with polysorbates 20 and 80 using ITC and DSC; they concluded that HSA interacted with polysorbates 20 and 80 with an association constant of approximately 10^3^ M^−1^ and a protein to polysorbate stoichiometry of 1:3. They also found that IgG did not interact with either polysorbate [28] for the one polysorbate grade studied. These two particular calorimetric techniques are often used for such studies for several reasons, firstly they are label-free meaning that the samples can remain unaltered for a more representative experiment, secondly they allow the determination of multiple parameters simultaneously. For example, DSC can produce results identifying changes in heat capacity and the thermal stability of a sample which are often used to infer the denaturation point of a protein, as well as changes in total enthalpy and Van’t Hoffs enthalpy leading to the determination of changes in Gibb’s free energy [29, 30].

Polysorbate 20 and 80 are commercially available at several levels of purity, known as grades, including regular grade (RG) and Super-refined™ (SR). Being of a higher grade, SR polysorbates differ from their RG counterparts with respect to their impurity tolerance, for example they contain a reduced peroxide content [31]. Few studies have compared different purities of polysorbate 20 and polysorbate 80 and their effects on the stability of biologics. One study by N. Doshi et al*.* investigated the effects of oxidative and reductive degradation of polysorbate 20 using two distinct purities, high purity (HP) and SR through degradation by enzyme interactions [32]. They concluded that SR polysorbate 20 was more prone to degradation than HP polysorbate 20 due to the increased oleate ester content and this led to an increased risk of aggregation.

This study considers two grades of polysorbate 20 and 80 to consider the effect of different polysorbates and grades on their interactions along with three model proteins ranging in size from 16 to 157 kDa to investigate whether surfactant interactions are protein-specific and/or polysorbate specific. Using ITC and DSC to characterise interactions utilising stoichiometry, binding constants, enthalpy changes and thermal stability will help formulators further understand how surfactants stabilise proteins, the nature of such interactions and ensure the most suitable surfactant is selected for each new biopharmaceutical formulation.

## Experimental

### Materials

Polyoxyethylene (20) sorbitan monolaurate (‘polysorbate 20’) and polyoxyethylene (20) sorbitan monooleate (‘polysorbate 80’) were donated by Croda Europe Ltd.: standard compendial grades, referred to as Tween™ 20 (BN:50,702) and Tween™ 80 (BN:49659A), and high purity grades referred to as Super Refined™ Polysorbate 20 (BN:0001814116) and Super Refined™ Polysorbate 80 (BN:0001779440). The Super Refined versions are distinct from the standard grades through their chemical composition including a low peroxide value (2.0 meq O_2_/Kg max.), limited formaldehyde (10 ppm max.), low residual EO (1 ppm max.), low 1,4-dioxane (5 ppm max.), low residual Na and K (5 ppm max.), low moisture (0.2% max.), decreased cellular irritation and microbial testing [31]. Three proteins were supplied by Sigma-Aldrich, UK, as lyophilised powders: immunoglobulin G (IgG) from human blood (> 99%), albumin from human serum (HSA) (> 96%) and β-lactoglobulin (β-Ig) from bovine milk (> 90%). These proteins were chosen to cover a range of molecular sizes from IgG (155 kDa) to HSA (65 kDa) to β-Ig (18 kDa). Potassium phosphate saline buffer of pH 7.4 was composed of 1.8 mM KH_2_PO_4_ (> 99%, Sigma-Aldrich, UK), 8.2 mM K_2_HPO_4_ (> 98%, Sigma-Aldrich, UK), 2.7 mM KCl (> 99%, Fisher Scientific, UK),140 mM NaCl (99.5%, Acros Organics, UK) and ultra-pure water (18.2 MΩ·cm).

### Methods

#### Isothermal titration calorimetry

Lyophilised protein powders were rehydrated by adding prepared buffer to the protein to achieve a concentration of 10 mg mL^-1^, then stored at 278 K for 72 h or until fully dissolved. Polysorbate concentrations chosen were 0.1 × and 1 × the CMC of each respective surfactant [15]. Due to the dilution of injection by the ITC, surfactant formulations were formulated at 10 times the desired concentration. ITC experiments were performed using a Microcal™ PEAQ-ITC from Malvern PANalytical. Prior to each experiment the sample cell and syringe were cleaned using 20% Contrad™ 70 detergent, rinsed with ultra-pure water and dried using methanol. Titration experiments were conducted at 303 K to ensure the experimental temperature was above laboratory ambient temperature. The reference cell was filled with ultra-pure water for all experiments. The 200 μL sample cell was filled with protein solution, and the 40 μL injection syringe was filled with surfactant solution and equilibrated at 278 K below the experimental temperature. The reference power was set to 10 µcal/sec. Experiments comprised of 14 injections of 2.5 µL at an injection speed of 0.5 µL/s. The time between injections was set long enough to allow the heat signals to return to baseline before the next injection. The solution in the reaction cell was continuously stirred at a speed of 500 rpm. A reference experiment of surfactant injected into buffer solution was conducted under identical conditions and subtracted from each run. Data were analysed using MicroCal PEAQ-ITC Analysis Software v1.41; the binding model was analysed using the one set of binding sites software present. Each experiment was repeated a minimum of three times to assess reproducibility.

#### Differential scanning calorimetry

Lyophilised protein powders were rehydrated by adding prepared buffer to the protein to achieve a concentration of 11 mg mL^-1^, then stored at 278 K for 72 h or until fully dissolved. Surfactant solutions were formulated at 10 times higher than the desired concentration (plus 10%) to account for dilution in formulation. Surfactant concentrations in experiments were 0.1 × and 1 × each surfactants respective CMC [15]. Samples were produced by dilution of a 10 mL protein solution with 1 mL of surfactant solution to produce the desired formulation. DSC experiments were conducted using a Microcal™ PEAQ-DSC machine from Malvern PANalytical. Prior to each experiment DSC cells were washed with 2% Decon™ 90 detergent and then rinsed with ultra-pure water. The DSC capillary cell volume was 250 µL for both reference and sample cells; for each experiment, the reference cell contained buffer solution. The sample cell contained a formulation of 10 mg mL^-1^ protein and the surfactant concentration being investigated. The scanning temperature range was set at 293—378 K at a rate of 333 K h^-1^. A reference scan of buffer solution was conducted under identical conditions and subtracted from each run. Data were analysed using MicroCal PEAQ-DSC Analysis Software v1.64.

## Results and discussion

### ITC

**Fig. 2:**
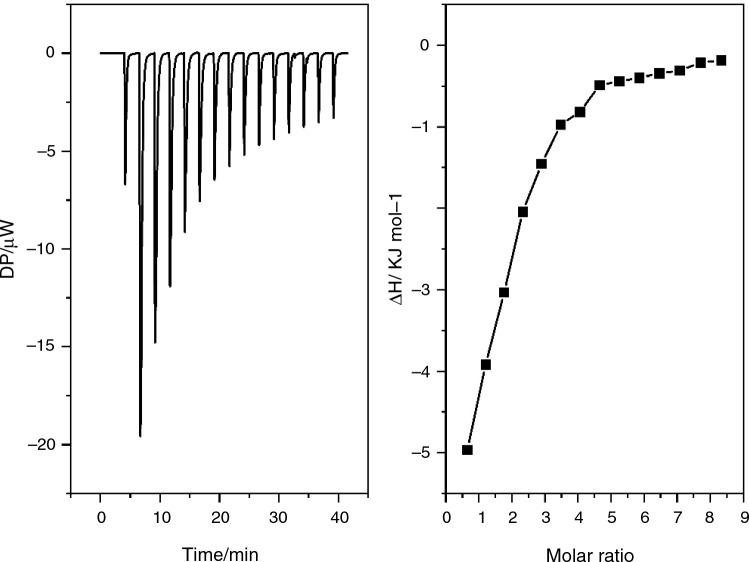
Left: ITC heat flow signal of the interaction of RG polysorbate 20 (1 × CMC) with β-Ig (0.54 mM) at 303 K. Right: reaction enthalpies as determined by peak integration of the ITC heat flow signal

Interactions between surfactants and proteins were first studied using ITC. ITC is a unique label-free method that can facilitate the calculation of multiple binding parameters simultaneously including stoichiometry (*n*), dissociation constant (*K*_d_), change in enthalpy (Δ*H*) and changes in Gibb’s free energy (Δ*G*) [33, 34]. Figure 2 depicts a typical ITC profile (RG polysorbate 20 with β-Ig at a surfactant concentration of 1 × the CMC) and reflects the general profile observed for all titrations performed. Results were analysed following subtraction of the reference reaction, which consists of heat generated by dilution, demicellisation and possible remicellisation of the surfactant. The first injection for each experiment generated a large exothermic peak, the enthalpy of each subsequent injection decreased as the experiment continued until a constant enthalpy change was observed.

A binding model considering one set of binding sites was used to analyse each experiment. Table 1 presents the results for experiments consisting of surfactant-β-Ig interactions at 1 × the CMC for each of the four surfactants, and Table 2 presents the results for surfactant-HSA interactions with the same experimental conditions. The data for the third protein analysed (IgG) are not shown as no binding event was observed and therefore no values for any of the parameters investigated (once the reference experiment was subtracted) were calculated. This is consistent with Garidel et al*.* who also found binding to be negligible for this protein when interacting with polysorbates 20 and 80 [28]. One possible explanation for this negligible binding is the lack of exposed hydrophobic regions on the protein reducing the available area for interaction by hydrophobic forces and van der Waal’s forces.

**Table 1:** ITC results for polysorbate-β-Ig interactions at 1 × CMC, CMC values extracted from the literature [15]. ^a^ = 2.4 mM, ^b^ = 1.5 mM, ^c^ = 2.5 mM, ^d^ = 2.1 mM

Protein	Surfactant	*n*	*K*_d_/µM	Δ*H*/kJ mol^−1^	Δ*G*/kJ mol^−1^
β-Ig	RG polysorbate 20^a^	1.65 ± 0.01	287 ± 52	−7.11 ± 0.53	−20.63 ± 0.45
	SR polysorbate 20^b^	1.83 ± 0.17	284 ± 99	−7.76 ± 1.22	−20.73 ± 0.84
	RG polysorbate 80^c^	1.42 ± 0.15	326 ± 21	−6.23 ± 0.31	−20.23 ± 0.19
	SR polysorbate 80^d^	1.31 ± 0.05	338 ± 43	−6.45 ± 0.24	−20.20 ± 0.09

Binding parameters investigated were stoichiometry (*n*), dissociation constant (*K*_d_), changes in enthalpy (Δ*H*) and changes in Gibb’s free energy (Δ*G*). Number of experimental repeats =  ≥ 3, error =  ± SD

**Table 2:** ITC results for polysorbate-HSA interactions at 1 × the CMC, CMC values extracted from the literature [15]

Protein	Surfactant	*n*	*K*_d_/µM	Δ*H*/kJ mol^−1^	Δ*G*/kJ mol^−1^
HSA	RG polysorbate 20^a^	3.01 ± 0.15	653 ± 60	−26.97 ± 1.60	−23.73 ± 3.94
	SR polysorbate 20^b^	3.01 ± 0.52	505 ± 74	−25.13 ± 5.85	−19.20 ± 0.46
	RG polysorbate 80^c^	2.50*	596 ± 21	−22.64 ± 0.70	−18.70 ± 0.08
	SR polysorbate 80^d^	2.29 ± 0.47	429 ± 51	−20.40 ± 5.35	−19.60 ± 0.28

^a^ = 2.4 mM, ^b^ = 1.5 mM, ^c^ = 2.5 mM, ^d^ = 2.1 mM. Binding parameters investigated were stoichiometry (*n*), dissociation constant (*K*_d_), changes in enthalpy (Δ*H*) and changes in Gibb’s free energy (Δ*G*). *Set at 2.50. Number of experimental repeats ≥ 3, error =  ± SD

Firstly, when comparing polysorbate grades and their interactions with β-Ig it was found that the binding parameters: *n*, *K*_d_, Δ*H*° and Δ*G*° displayed little difference between purity grades (with almost all values falling within error limits). β-Ig interactions with RG and SR polysorbate 20 produced stoichiometry values of 1.65 and 1.83, respectively, and interactions with RG and SR polysorbate 80 produced lower values of 1.46 and 1.31, implying that the ratio of surfactant to protein binding is approximately 3:2. A direct comparison with published literature is challenging as analysis of this combination of polysorbate and protein using ITC is not commonly assessed; however, J.Chen et al. found interactions occurring at a ratio of 4:1 (surfactant to protein) at an oil–water interface [35]. This value is more than has been observed in this study; however, the nature of the interface may have led to the difference in the activity of both the surfactant and the protein given their amphiphilic natures. The ratio of this activity is thought to arise from the structure of the protein; β-Ig is a small protein with a molecular mass of 18.4 kDa and possesses small regions of hydrophobic amino chains that are exposed to the environment [36]. It is these conditions that allow surfactants to interact with proteins and in ITC the strength of these interactions is reflected in the dissociation constant. Dissociation constant values for all four surfactants were within the range 259 to 337 µM. These *K*_d_ values contrast with previously published literature by Taheri-Kafrani et al*.* who studied β-Ig binding interactions with Triton X-100, another non-ionic surfactant [37] and found that at molar ratios similar to those used in this study Triton X-100 bound to β-Ig with a *K*_d_ value of 0.08 µM. This value is significantly smaller than β-Ig interactions in this study and indicates far stronger binding between Triton-X and β-Ig compared with polysorbates. This discrepancy could be the result of differences between the surfactants themselves as Triton X-100 is a much smaller molecule (with a molecular mass half that of polysorbates 20 and 80), which could make binding to various binding sites located on β-Ig easier. As it is desirable for a surfactant used in formulations to have a higher *K*_d_ value and therefore be less likely to bind to protein molecules, then it can be postulated that polysorbates are more suited for biopharmaceutical formulations than Triton-X.

Next, when considering the changes in enthalpy associated with the surfactant–protein interactions, Δ*H* values of both grades of polysorbate 20 were around −7.4 kJ mol^−1^ and polysorbate 80 around −6.2 kJ mol^−1^, with all four surfactants within the error limits. Again, a direct comparison to published literature is difficult when considering this combination of surfactants and protein although Jung *et.al.* studied β-Ig interactions with sodium dodecyl sulphate (SDS) [38] and observed large exothermic peaks that reduced as binding sites became saturated, similar to the results produced in this study. It should be noted that in their work determination of *K*_d_ was made unfeasible because of the nature of the micelles. Overall, it can be concluded that β-Ig and the four polysorbates interacted with a measurable ratio that the binding affinity of β-Ig to polysorbates is weaker than other surfactants seen in the literature and despite this the interaction is thermodynamically favourable. This is further established in the Δ*G* values for all experiments, which were observed to be around -20 kJ mol^−1^ as all free energy values were negative; thus, binding is confirmed to be spontaneous and thermodynamically favourable. A contributing factor to the free energy values could be a consequence of the surfactants ability to reduce surface tension. While this study seeks to observe the surfactant–protein interactions, polysorbates will still reduce the overall surface tension within each experiment and it has been demonstrated in published literature that polysorbate 20 reduces surface tension more than polysorbate 80 [39]. These interactions are governed by hydrogen bonding, hydrophobic interactions and van der Waals forces [40].

Following β-Ig a similar set of experiments were undertaken for HSA as summarised in Table 2.

As for HSA, initially focusing on polysorbate 20 with regard to stoichiometry, Table 2 indicates polysorbate 20 displayed little difference (within error limits) between surfactant purities with a surfactant–protein binding ratio of 3:1 in both cases. However, the dissociation constant identified SR polysorbate 20 binds to HSA with a slightly higher affinity than RG polysorbate 20, as these two values are not within error limits. Interestingly, both values are larger than all four binding values with β-Ig indicating polysorbates bind with a higher affinity to β-Ig than for HSA. Compared with published literature, *K*_d_ values are similar to that of Garidel et al*.* (625 µM) when titrating polysorbate 20 into HSA in different buffer conditions at a lower temperature (298 K) [28]. However the values presented in this work are higher than those reported by Chou et al*.* (*K*_d_ = 135 µM) [41]; this difference is most likely due to the modifications made to human albumin by genetically fusing it with human growth hormone to produce Albutropin™.

**Fig. 3:**
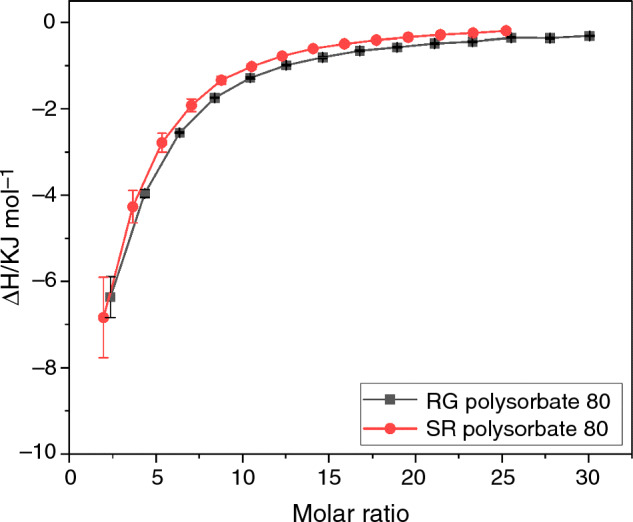
Integrated heat plots of ITC injections for RG polysorbate 80 (1 × CMC) and SR polysorbate 80 (1 × CMC) into HSA (0.16 mM) at 303 K. Number of experimental repeats = 3

With regard to polysorbate 80 grades, initially RG polysorbate 80 produced a binding ratio of approximately 1:1, whereas SR polysorbate 80 produced a value a little over 2:1. However it can be seen in Fig. 3, when comparing the integrated heat plots of both grades of polysorbate 80, the midpoint of the integrated heat curves appears to occur at a similar molar ratio for each experiment, suggesting a similar value for *n* would be derived, yet this was not the case. This difference in reported *n* could be the result of limits of the binding model used to accurately differentiate between certain sets of data. Compared with published values, we can see that Garidel et al*.* produced an *n* value of between 1 and 3 [28]; therefore, in this study it was decided to set the *n* value in the analysis software to 2.5 which produced similar Δ*G* values (around -18 kJ mol^−1^) to published literature. Prior to setting *n* at 2.5 the discrepancy in vastly different *n* values meant that the Δ*H* and *K*_d_ values were also initially not comparable with published literature, further justifying caution to be taken when analysing such data. In general, results for HSA suggest that the reaction occurred at a polysorbate-specific ratio with a weaker binding affinity (i.e., beyond error limits) than seen for β-Ig, yet the polysorbate–protein interaction remains a thermodynamically favourable event in all cases.

Data for surfactant–protein interactions at concentrations 0.1 × the CMC have not been included as calculated values implied suspected inaccuracies in ITC binding models. As previously discussed in several published studies, ITC binding models are limited in accuracy by the so-called *c* value [42, 43]. This value is typically recommended to be between 5 and 50, while in this study the *c* value (particularly for 0.1 × CMC experiments) fell below this range, inferring results are less reliable. In order to increase the *c* value in these experiments the concentration of the titrant would have to be increased in the sample cell (Eq. 1). The solubility limits of the protein samples used in this study varied between 30 and 50 mg mL^-1^, using the maximum solubility for each individual protein would still not have placed the *c* value inside the ideal range and so a standard concentration was used that it is still pharmaceutically relevant and easy to compare between samples [44].

1 $$c=n \times \left[\text{Titrant }(\text{M})\right]/{K}_{\text{d}}$$

Equation for calculating the *c* value of an ITC system.

Interpretation is therefore inherently more unreliable and less reproducible, as the concentration of the surfactant, which can be thought of as the ligand in this experiment, decreases so does binding saturation leading to further increases in inaccuracy (which is the case for experiments using polysorbate concentrations of 0.1 × the CMC). This also provides further justification of the need to fix the stoichiometry in Table 2 for RG polysorbate 80 which initially produced a much larger value for *K*_d_ and Δ*G*. It is suggested by this study that despite saturation occurring in the experiment, the initial values produced are a consequence of the low *c* value causing the binding model to compensate for Δ*H* inaccuracy by increasing the *K*_d_ value in order to reach the Δ*G* value seen. Based on the low *c* values obtained in some reactions in this study, an orthogonal method was employed to validate the results, namely DSC.

### DSC

DSC experiments are often conducted to evaluate the thermal stability of proteins [45]. During a DSC experiment a protein will unfold as a result of temperature-induced denaturation; this will present as a peak in the DSC graph, the mid-point of which is measured and referred to as the *T*_m_. As thermal stability of a protein is increased so does the temperature at which *T*_m_ occurs [21, 23, 25], all three proteins were analysed using DSC to determine thermal profiles for unfolding in the presence of all four polysorbates.

**Fig. 4:**
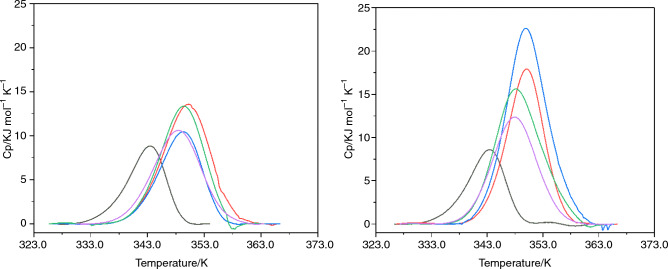
DSC profiles for β-Ig at 0.1 × the CMC (left) and 1 × the CMC (right). β-Ig without surfactant (black) β-Ig with; RG polysorbate 20 (red), SR polysorbate 20 (blue), RG polysorbate 80 (green) and SR polysorbate 80 (purple)

Firstly, it was found that all four surfactants considered at both concentrations (0.1 × and 1 × the CMC), did increase the *T*_m_ of β-Ig implying improved protein stability (Fig. 4).

At 0.1 × the CMC all four surfactants increased the average *T*_m_ by 5 (± 1) K (Table 3). This trend continued when the concentration of surfactants was increased to 1 × the CMC, at this concentration both grades of polysorbate 20 increased the *T*_m_ by 4 K (Table 4) and both purities of polysorbate 80 increased the *T*_m_ by 6 K. However, when considering the standard deviation in this data there is almost no difference between *T*_m_ values. These results imply that the presence of surfactant increased stability, but that increasing surfactant concentration did not cause an increase in thermal stability in relation to *T*_m_.

**Table 3:** DSC results for polysorbate-β-Ig interactions at 0.1 × the CMC, CMC values extracted from the literature [15]

Protein	Surfactant	Total area/kJ mol^-1^	*T*_m_/K
β-Ig	None	78.4 ± 6.6	343.6 ± 0.2
	RG polysorbate 20^a^	123.5 ± 14.5	347.7 ± 2.4
	SR polysorbate 20^b^	107.3 ± 11.7	347.7 ± 1.4
	RG polysorbate 80^c^	121.5 ± 3.5	349.7 ± 0.2
	SR polysorbate 80^d^	117.0 ± 6.0	349.5 ± 0.9

^a^ = 2.4 mM, ^b^ = 1.5 mM, ^c^ = 2.5 mM, ^d^ = 2.1 mM. Number of experimental repeats ≥ 2, error =  ± SD

**Table 4:** DSC results for polysorbate-β-Ig interactions at 1 × the CMC, CMC values extracted from the literature [15]

Protein	Surfactant	Total area/kJ mol^-1^	*T*_m_/K
β-Ig	None	78.4 ± 6.6	343.6 ± 0.2
	RG polysorbate 20^a^	149.0 ± 0.0	349.9 ± 0.2
	SR polysorbate 20^b^	177.5 ± 31.0	350.0 ± 0.3
	RG polysorbate 80^c^	139.5 ± 20.5	348.3 ± 0.4
	SR polysorbate 80^d^	123.0 ± 2.0	347.9 ± 0.2

^a^ = 2.4 mM, ^b^ = 1.5 mM, ^c^ = 2.5 mM, ^d^ = 2.1 mM. Number of experimental repeats ≥ 2, error =  ± SD

When looking at the values presented in Tables 3 and 4 an increase in the total area compared with protein alone can be observed. This is of considerable importance as there is a direct relationship between the total area and concentration of folded protein [26]. The total enthalpy of transition in protein analysis is directly proportional to the concentration of native folded proteins contributing to the transition; this means that a larger enthalpy transition means more protein is present in its native conformation at the beginning of the experiment. In this study the concentration of protein was kept constant for each experiment, meaning in the case of β-Ig in Fig. 4, that all experiments containing surfactants also possessed a larger concentration of folded protein relative to protein without surfactant. This would suggest that the surfactants are either preventing unfolding of native proteins or they are chaperoning the refolding of unfolded proteins or a combination of the two processes [46]. This effect of polysorbates has been demonstrated by Chou et al*.* who used polysorbates to infer thermal stability to Albutropin [47]. Results for β-Ig are unique and do not compare easily to published literature, for example Kresheck looked at the denaturation of β-Ig in the presence of different non-ionic surfactants whereby the surfactants did not infer any thermal stability [48]. Magdassi et al*.* also studied β-Ig thermal stability but in the presence of SDS and DTAB and found that these surfactants did infer thermal stability with an increase in the *T*_m_ of around 2 K, which is surprising as they are known protein denaturants [49].

**Fig. 5:**
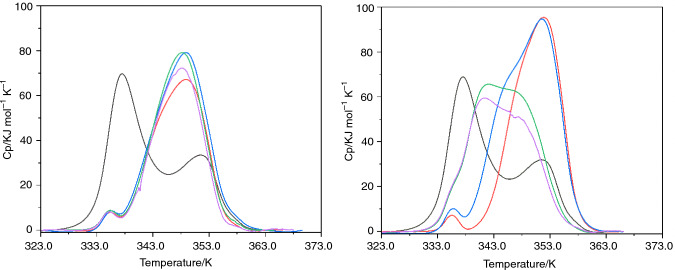
DSC profiles for HSA at 0.1 × the CMC (left) and 1 × the CMC (right). HSA without surfactant (black) HSA with; RG polysorbate 20 (red), SR polysorbate 20 (blue), RG polysorbate 80 (green) and SR polysorbate 80 (purple)

For HSA all four surfactants did not increase the *T*_m_ beyond the error limits. Figure 5 displays typical profiles for both concentrations used (0.1 × and 1 × the CMC).

HSA without surfactant produced two distinct peaks, peak 1 at 338 K and peak 2 at 349 K; the first peak compares well with the DSC profile reported by Picó [22]. In this study when HSA was exposed to polysorbates at 0.1 × the CMC the DSC profiles changed notably, the first large peak at 335 K appeared to reduce in size and the peak at 348 K increased (Table 5).

**Table 5:** DSC results for polysorbate-HSA interactions at 0.1 × the CMC, CMC values extracted from the literature [15]

Protein	Surfactant	Total area/kJ mol^-1^	*T*_m_ 1/K	*T*_m_ 2/K
HSA	None	1039.5 ± 200.5	337.0 ± 1.2	349.4 ± 0.1
	RG polysorbate 20^a^	863.0 ± 75.0	337.6 ± 2.0	349.9 ± 1.8
	SR polysorbate 20^b^	904.5 ± 20.5	338.6 ± 2.1	349.6 ± 1.3
	RG polysorbate 80^c^	870.5 ± 0.5	340.5 ± 3.7	348.6 ± 0.6
	SR polysorbate 80^d^	808.0 ± 29	339.9 ± 4.8	348.3 ± 0.6

^a^ = 2.4 mM, ^b^ = 1.5 mM, ^c^ = 2.5 mM, ^d^ = 2.1 mM. Number of experimental repeats ≥ 2, error =  ± SD

The observed changes in DSC profiles could have multiple explanations as HSA has multiple amino acid chains that constitute the protein and each of these chains could respond differently to the temperature increase, as well as their binding activity with polysorbates. Firstly, in the case of the peak at 338 K, it could be an increase in thermal stability which results in a temperature shift to that of the peak at 348 K. However, this does not account for the small enthalpy peak remaining.

Another explanation for the change in the DSC peaks is that as HSA has three amino acid chains that contribute to the total enthalpy change [50], the first peak present in Fig. 5 at 338 K consists of two events one of which is being concealed by the other. When HSA is formulated with polysorbates, the two amino acid chains responsible for the two events respond differently. The first of which is stabilised against the increase in temperature and presents as an increase in *T*_m_. The second amino acid chain does not respond to the addition of polysorbates and does not undergo an increase in *T*_m_, leaving it exposed in a similar manner to that seen without polysorbates. This explanation can also be applied to formulations which possess polysorbates at 1 × the CMC (Table 6) as the shift from one peak into another seemed to be incomplete and caused convolution of all peaks present.

**Table 6:** DSC results for polysorbate-HSA interactions at 1 × the CMC, CMC values extracted from the literature [15]

Protein	Surfactant	Total area/kJ/mol	*T*_m_ 1/K	*T*_m_ 2/K
HSA	None	1039.5 ± 200.5	337.0 ± 1.2	349.4 ± 0.1
	RG polysorbate 20^a^	1089.5 ± 90.5	341.3 ± 5.6	350.3 ± 1.8
	SR polysorbate 20^b^	1180.0 ± 0.0	349.4 ± 2.4	348.6 ± 3.3
	RG polysorbate 80^c^	988.5 ± 11.5	338.4 ± 2.8	344.0 ± 2.2
	SR polysorbate 80^d^	856.0 ± 8.0	338.0 ± 2.4	344.2 ± 2.1

^a^ = 2.4 mM, ^b^ = 1.5 mM, ^c^ = 2.5 mM, ^d^ = 2.1 mM. Number of experimental repeats ≥ 2, error =  ± SD

The change in profile for *T*_m_ 1 and *T*_m_ 2 could be a consequence of the increased concentration of surfactant resulting in a weaker stabilisation of the first peak or a reduction in stabilisation of the second peak. This convolution of peaks also makes determination of the total enthalpy change difficult as it seems to decrease with the addition of polysorbate 80; this again could be because the polysorbate is interacting differently with each component of the HSA molecule protecting some amino acids chains as they did for β-Ig but causing the unfolding of other chains before the experiment had even begun. It should be stated that DSC profiles for both proteins depend on many factors, including buffer composition, making comparison to published literature difficult [51].

**Fig. 6:**
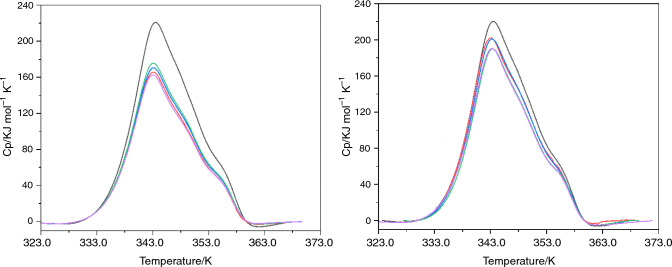
DSC profiles for IgG at a CMC concentration of 0.1 x (left) and 1 x (right) IgG without surfactant (black) IgG with; RG polysorbate 20 (red), SR polysorbate 20 (blue), RG polysorbate 80 (green) and SR polysorbate 80 (purple)

The third protein to be studied using DSC was IgG; Fig. 6 displays DSC profiles for IgG interactions with surfactants.

In all cases, within error limits, there was no increase in the *T*_m_ of IgG in the presence of any of the four surfactants studied. There was also no increase in total change in enthalpy in relation to the total area, i.e., these data are consistent with the accompanying ITC results implying no interaction. In a previously published study [28], a minor increase of only 0.3 K was found for a similar system which adds further evidence that no interactions had occurred between IgG and polysorbates 20 and 80 of either grade in this study. The lack of interactions observed in both the ITC and DSC experiments may be a consequence of the proteins size as it is much larger than the other proteins analysed in this study. Another explanation relates to the isoelectric point of the proteins studied; HSA and β-Ig possess isoelectric points (pI ~ 5) that are different to IgG (pI ~ 7) and therefore could be causing electrostatic interactions with surfactants [52–54]. However, in all cases the pH is higher than the pI and more importantly, polysorbates are non-ionic surfactants and therefore electrostatic interactions are unlikely. A more likely explanation is differences in amino acid composition as IgG has a structure that consists primarily of beta-sheets as opposed to β-Ig and HSA that also contain alpha-helices. Further evidence to this hypothesis exists in a published study by Fan *et.al.* who investigated the interaction of biosurfactants and proteins [55]. They discovered that the biosurfactant employed caused changes in the proteins secondary structure, namely a reduction in the number of beta-sheets present, but they also observed an increase in the number of alpha-helices present, which in turn increased the *T*_m_ for the protein β-glucoside. While beta-sheets do not seem to be responsible for changing *T*_m_ in a protein, they may interact by another mechanism that does result in the change in enthalpy observed in this study. The absence of different results between the polysorbate quality grades further confirms that these observations are a direct reflection of protein–surfactant interactions, and not a result of interactions with minor impurities that may be present in certain lower polysorbate qualities.

## Conclusions

This study sought to investigate differences in thermodynamic activity of polysorbates 20 and 80 and compare the effects of different grades on interactions with model proteins. ITC experiments demonstrated that all four polysorbates bound to β-Ig and HSA, but no activity was shown in relation to IgG. However, results were only considered reliable at surfactant concentrations of 1 × CMC, below which results relied upon non-reproducible interpretation and required an additional technique. DSC demonstrated similar results, interactions that conferred thermal stability occurred in β-Ig and HSA for all surfactants and both concentrations considered. For β-Ig an increase in total enthalpy suggested an increase in initial concentration of natively folded protein. Therefore, surfactants can prevent unfolding or chaperone refolding of proteins, not only increasing *T*_m_ but also producing formulations that are more resistant to unfolding. Using ITC and DSC has facilitated analysis of multiple parameters and observation of surfactant–protein interactions that cannot be seen with other techniques. This study is the first of its kind to have compared surfactant interactions with the three proteins analysed in this study. Formulation scientists now have more information with regard to polysorbate grade effects that will allow them to choose the most suitable type and grade of polysorbate for new medicines.
